# The effect of a planned lactation education program on the mother’s breastfeeding practice and weight gain in low birth weight infants: a randomized clinical trial study

**DOI:** 10.1186/s12884-022-04810-z

**Published:** 2022-06-13

**Authors:** Afsar Omidi, Sahar Rahmani, Roya Amini, Manoochehr Karami

**Affiliations:** 1 Department of Community Health Nursing, School of Nursing and Midwifery, Hamadan University of Medical Sciences, Hamadan, Iran; 2 Community Health Nursing, School of Nursing and Midwifery, Hamadan University of Medical Sciences, Hamadan, Hamadan, Iran; 3 Department of Community Health Nursing, School of Nursing and Midwifery, Chronic Diseases (Home care) Research Center, Hamadan University of Medical Sciences, Shahid Fahmideh Avenue, Hamadan, Iran; 4 Department of Epidemiology, Shahid Beheshti University of Medical Sciences, Tehran, Iran

**Keywords:** Breast feeding, Breast milk, Education, Infant, Low birth weight, Weight gain

## Abstract

**Background:**

Low birth weight (LBW) infants are more prone to possible growth disorders, and their mothers need more specific education sessions regarding breastfeeding practice. This study aimed to investigate the effect of a planned lactation education program on the mother’s breastfeeding practice and weight gain in LBW infants.

**Methods:**

A randomized clinical trial study was conducted on 80 mother-LBW infant dyads admitted to a gynecology and obstetrics hospital. The participants were selected randomly and divided into an experimental group and a control group, each with 40 mothers. Information on LBW infants’ weight and the mothers’ breastfeeding practice was collected using a questionnaire at birth. Then, a planned lactation education program was implemented in the experimental group in two sessions in the hospital and three 20-minute sessions in comprehensive health centers. Finally, the weight of 14–15 day-old and two-month-old LBW infants and the mothers’ breastfeeding practice for 14–15 day-old LBW infants in the two groups were recollected and analyzed using SPSS software version 16.

**Results:**

Comparing the LBW infants’ weights and mothers’ breastfeeding practice revealed no statistically significant difference between the two groups pre-intervention. However, significant differences were observed between the two groups post-intervention in terms of weight gain in the LBW infants over 14–15 days and two months of age (F = 4720.6, *p* < 0.001) and the mothers’ breastfeeding practice for 14-15-day-old infants (*p* < 0.001).

**Conclusions:**

Given the positive impact of lactation education on the mother’s breastfeeding practice and LBW infants’ weight, planned lactation education courses should be applied for LBW infants’ mothers.

**Trial registration:**

This study was retrospectively registered in the Clinical Trial Registration Center of Iran, with the code: IRCT20120215009014N421 on 14/04/2022.

## Background

Low birth weight (LBW) significantly indicates an infant’s survival and intrauterine growth [1]. It is estimated that LBW accounts for 15–16% of total births globally [2, 3]. The World Health Organization has defined LBW as a birth weight of less than 2500 gr [4]. LBW infants are more likely to suffer from short- and long-term physical, mental, and social disabilities [5, 6].

LBW infants are at greater risk of complications, adverse outcomes, and early mortality, and they need more special healthcare programs, such as feeding programs, than other babies [7]. They often feed slowly and require multiple rest times; thus, mothers need to spend much time daily breastfeeding LBW infants [8]. Furthermore, LBW infants should receive enough calories to gain 10 to 20 g per day [9]. Thus, LBW infants are more prone to possible growth disorders and should obtain adequate energy and nutrition [10].

As LBW infants have high nutritional requirements, they must feed on their mother’s own milk (MOM), which significantly affects their weight and length [11]. Furthermore, exclusive breastfeeding up to six months of age or MOM is recommended for these infants [12] due to advantages such as decreased risk of lateonset sepsis, necrotizing entercolitis, reduced feed intolerance, and appropriate neurodevelopmental status [13].

Despite the importance of breastfeeding, some studies show that early initiation of breastfeeding and exclusive breastfeeding are low, especially in LBW infants [14, 15]. LBW infants’ mothers need to promote their ability to breastfeed and ensure exclusive breastfeeding continuance; thus, they require counseling and additional training intervention regarding breastfeeding practice, especially for their infants’ first 28 days of life [15, 16]. In this regard, healthcare workers, i.e., nurses, have an essential role in encouraging mothers to initiate and accomplish breastfeeding [12]. Meanwhile, healthcare workers should frequently assess the LBW infants’ weight and record their weight gain because of the significant relationship between weight gain and subsequent health outcomes of LBW [17, 18]. Moreover, healthcare workers should provide continued care and apply education programs to help average infant growth and development [17].

Based on previous studies, early breast milk expression, rooming-in, skin-to-skin contact, infant test weighing, and the reduced use of pacifiers are significant factors associated with exclusive breastfeeding, especially in LBW infants [19, 20]. To improve breastfeeding practice and, consequently, weight gain in LBW infants, mothers need continued encouraging education through strategies, such as combining hospital-based [19] and community-based training [21, 22].

In Iran, the number and content of training sessions held for LBW infants’ mothers regarding breastfeeding practice are the same as those held for other infants. However, these mothers need more specific training sessions. As breastfeeding is a continuous behavior, hospital training sessions could be followed by specific community-based training. Thus, this study aimed to investigate the effect of a planned lactation education program on the mother’s breastfeeding practice and weight gain in LBW infants.

## Methods

### Trial design

A randomized clinical trial pretest-posttest design was conducted on mother- LBW infant dyads in a gynecology and obstetrics hospital and comprehensive health centers affiliated with Hamadan University of Medical Sciences, Iran, in 2016 (Research number: 9,409,034,759, IRCT20120215009014N421). A Planned lactation education was implemented for the mothers in the experimental group over five training sessions in addition the routine education by one of the researcher; however, the control group (socio-demographically homogenous to the experimental group) received the routine education program. The main parameters concerned in this study included the mothers’ breastfeeding practice, and the LBW infants’ weight gain.

### Participants

In this study, 80 mothers of LBW and their infants participated. The inclusion criteria for infants were birth weight between 1500 and 2500 g, completed weeks of gestation (more than 37 weeks), exclusive breastfeeding, and lack of diseases, infections, and history of hospital admission due to diseases. The inclusion criteria for mothers were literacy, singleton pregnancy, no prohibition due to disease or medications preventing breastfeeding, no participation in breastfeeding training classes for LBW, and residency in Hamadan City. Mothers changing their residency, becoming sick during the study, or stopping breastfeeding and infants receiving supplementation with formula were also excluded.

### Intervention

 After approving the study in the ethics committee, the researchers referred to the gynecology and obstetrics hospital. The researchers explained the study’s objectives to the mothers, and written consent forms were collected from them. The researchers completed the demographic data questionnaire of the parents and their LBW infants. Furthermore, the researchers evaluated the LBW infants’ birth weight and the mother’s breastfeeding practice.

The intervention for the experimental group included routine education and planned lactation training containing five 20-minute face-to-face teaching sessions, with two sessions being held during the mothers’ hospitalization and at discharge time and three sessions being held in the comprehensive health centers at 5, 14–15, and 60 days after the infants’ birth. Furthermore, the mothers received a CD and a written instruction booklet regarding breastfeeding and infant weight gain at the first session.

The training content emphasized the importance of initiating breastfeeding and exclusive breastfeeding for LBW infants, the number and time duration of each breastfeeding per day, the significant skin contact with LBW infants, and significant differences between LBW infants and other infants regarding breastfeeding. Moreover, the mothers were trained regarding correct breastfeeding, i.e., how to hug the baby, provide proper alignment for breastfeeding, make skin-to-skin contact, place the nipple in the baby’s mouth, and spend more time for breastfeeding. The education program also focused on proper weight gain of LBW infants.

After the discharge, all the mother-LBW dyads were followed up in comprehensive healthcare centers, and the researchers controlled weight gain in the infants and answered the mothers’ questions. Furthermore, feedbacks were given to the mothers about weight gain according to the infants’ growth chart. Finally, the infants’ weights were recollected and documented 14–15 days and 60 days after birth; however, the mother’s breastfeeding practice data were recollected 14–15 days after birth.

The control group attended a routine education program, including one 10-minute session held in hospital and two 15-minute sessions held in the comprehensive health centers with a similar educational content provided to mothers in the experimental group, 14–15 days and 60 days after the infants’ birth. However, they did not receive any CDs or written booklets. It is noteworthy that as the number of sessions was greater and the duration of each session was longer in the experimental group compared to the control group, it was possible for the researchers to spend more time answering mothers’ questions, precisely monitor breastfeeding performance, and improve positioning and attachment. As a result, mothers in the experimental group received more careful supervision and more supportive encouragement.

### Outcome measures

The data were collected using four instruments: Demographic data of the parents, demographic data of the LBW infants, the LBW infants’ weight, and the mothers’ breastfeeding practice.

The parents’ demographic data included mothers’ age, education level of parents, parents’ occupation, family income, and family residency.

The infants’ demographic data were assessed by a questionnaire including gestational age, sex, birth rank, and type of delivery.

The LBW infants’ weight was measured by the researchers according to the guidelines [23] using a balanced Seca scale. The LBW infants’ weight was measured at birth, 14–15 days, and two months after birth.

The researchers applied the checklist used by Ahmadi et al. [24] at birth and 14–15 days later to evaluate the mother’s breastfeeding practice. This checklist comprises 17 questions regarding the correct method of breastfeeding (e.g., infant’s body being in line with the mother’s body, infant’s body having close contact with the mother’s body, the majority of the areola being in the infant’s mouth, gentle and deep sucking of the breast by the infant, and no pain on the mother’s nipple while breastfeeding). The researcher observed breastfeeding practice and gave a score of “1” for the correct performance of each item and “0” otherwise. Thus, the minimum and maximum scores ranged from 0 to 17.

The validity of the mother’s breastfeeding practice checklist was confirmed using qualitative content validity. The reliability of the breastfeeding practice checklist was determined by a pilot study carrying out on 20 LBW infants’ mothers. Intra-class coefficients were ≥ 0.92, indicating acceptable reliability. Before each weight measurement time, a standard 500 g weight was used to determine the infant weighing scale’s reliability.

### Sample size

The sample size was estimated to be 37 people for weight gain based on the estimated variance of 3.1 and a minimum significant difference of 2 between the two groups. The sample size was also estimated to be 35 people for breastfeeding practice based on the estimated variance of 5 and a minimum significant difference of 3.3 between the two groups (based on a pilot study). The confidence level of the test and the test power was considered 95% and 80%, respectively. The sample size of each group was enhanced to 43 to increase the power of statistical tests.

### Randomization

In this study, eligible mothers and their LBW infants randomly assigned through the block randomization into two groups with 40 mother-LBW infants dyads in experimental (receiving a planned lactation training and routine education) and control (receiving routine education) groups. For this purpose, researchers prepared four sheets of paper, writing on two sheets the name of the experimental and on the other two sheets the name of the control. The paper sheets were folded, placed in a container, and randomly drawn one at a time for each mother without replacement until all four sheets are drawn. The four paper sheets were then placed back into the container, and this action repeated until the sample size were reached.

### Statistical methods

The collected data were analyzed at a 95%confidence interval using the Kolmogorov-Smirnov test, paired samples t-test, independent two-sample t-test, and repeated measures ANOVA in SPSS software version 16.

## Results

**Fig. 1:**
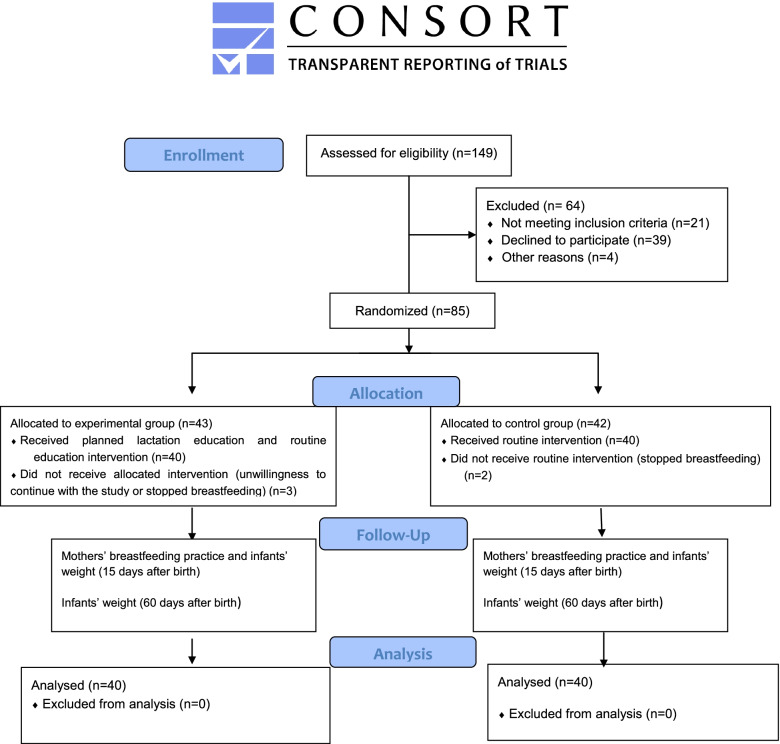
Flow chart of the study

A total of 85 samples were included in this study. In the experimental group, 3 people were excluded (2 due to stopping breastfeeding and 1 for unwillingness to continue with the study). Also in the control group, 2 people were excluded (for stopping breastfeeding) (Fig. 1).

According to the findings presented in Table 1, most of the mothers’ age range in the experimental (37.5%) and control groups (35%) were 21–25 and 26–30 years old, respectively. The education of most of the mothers in the experimental (55%) and control (62.5%) groups were diplomas. Most of the mothers in the experimental (72.5%) and control (67.5%) groups were housewives. Moreover, most of the LBW infants’ fathers in the experimental (45%) and control (47.5%) groups had an academic education, and a majority of them in the experimental (42.5%) and control (45%) groups were employed. A majority of families in the experimental (47.5%) and control (52.5%) groups had a low-income level. Moreover, most of the families in the experimental (55%) and control (67.5%) groups had three family members. According to the comparative analyses, the two groups had no significant difference in terms of the aforementioned variables (*P* > 0.05).

Moreover, most of the LBW infants in the experimental (57.9%) and control (67.6%) groups were boys (*p* = 0.387), and a major of infants in the experimental (55.3%) and control (63.2%) groups had a vaginal delivery (*p* = 0.368).

**Table 1:** Comparison of demographic characteristic between the two groups of LBW infant s’ parents

Variable	Levels of variable	Experimental group	Control group	Statistical test ^a^
		N (%)	N (%)	
Mothers’ age (year)	15–20	5 (12.5)	6 (15)	*x*^*2*^ *= 4.004* *p* = 0.541
	21–25	15 (37.5)	10 (25)	
	26–30	9 (22.5)	14 (35)	
	31–35	6 (15)	6 (15)	
	36–40	5 (12.5)	4 (10)	
Mothers’ education	Elementary	5 (12.5)	4 (10)	*x*^*2*^ *= 2.007* *p* = 0.571
	Diploma	22 (55)	25 (62.5)	
	Academic education	13 (32.5)	11 (27.5)	
Mothers’ occupation	Housewife	29 (72.5)	27 (67.5)	*x*^*2*^ *= 0.682* *p* = 0.877
	Employed	11 (27.5)	13 (32.5)	
Fathers’ education	Elementary	8 (20)	6 (15)	*x*^*2*^ *= 2.365* *p* = 0.669
	Diploma	14 (35)	15 (37.5)	
	Academic education	18 (45)	19 (47.5)	
Fathers’ occupation	Unemployed	3 (7.5)	3 (7.5)	*x*^*2*^ *= 2.987* *p* = 0.810
	Worker	7 (17.5)	8 (20)	
	Self-employed	13 (32.5)	11 (27.5)	
	Employed	17 (42.5)	18 (45)	
Family income	Low (Less than 50$)	19 (47.5)	21 (52.5)	*x*^*2*^ *= 0.750* *p* = 0.832
	Moderate (50–199 $)	14 (35)	12 (30)	
	High (200$ and more)	7 (17.5)	7 (17.5)	
Number of family members	Three	22 (55)	27 (67.5)	*x*^*2*^ *= 0.388* *p* = 0.143
	Four	13 (32.5)	10 (25)	
	Five	5 (12.5)	3 (7.5)	

^a^ Chi-square test

As shown in Table 2, there were no significant differences between LBW infants’ weight in the experimental and control groups before the education program (*p* > 0.05). However, after the education program, the mean score of the LBW infants’ weight significantly increased in each of the experimental (*p* < 0.001*)* and control groups (*p* < 0.001) over 14–15 days and two months. Even though, repeated measures analysis of variance showed significant differences regarding LBW infants’ mean weight gain in the experimental group compared to the control group over14-15 days and two months (*p* < 0.001)

**Table 2:** Comparison of the weight gain at 14–15 day and two-month-old LBW infants in two groups

Infants’ weight (gr)	Birth	14-15-day old	Two month old	Repeated Measures ANOVA	Repeated Measures ANOVA
	Mean ± SD	Mean ± SD	Mean ± SD		
Experimentalgroup	2297.5 ± 62.39	2565.6 ± 53.16	3606.3 ± 61.45	*F*= 4720.6 *P< 0.001*	F = 9467.5 *P < 0.001*
Controlgroup	2282.5 ± 51.90	2348.9 ± 25.80	3270.8 ± 64.90	*F*= *4969.25* *P*< 0.001	
Independentt-test	*P *= 0.246	*P *< 0.001	*P*< 0.001		

According to the findings presented in Table 3, the mothers’ breastfeeding scores had no significant difference in the experimental and control groups before the education program (*p* > 0.05). However, it significantly increased in the experimental group comparing the control groups after the lactation training program (*p* < 0.001).

**Table 3:** Comparison of mothers’ breastfeeding practice of LBW infants before and after education in two groups

Breastfeeding scores	Range	Before education	After education	Statistical tests^a^
		**Mean ± SD**	**Mean ± SD**	
Experimental group	0–17	4.42 ± 0.07	11.28 ± 0.55	*p < 0.001*
Control group	0–17	4.43 ± 0.05	8.29 ± 0.06	*p* < 0.001
*P* value^b^		*p =* 0.45	*p* < 0.001	

^a^ Independent t-test ^b^ Paired t-test

## Discussion

The study results showed that following the education program, the weight of LBW infants in the experimental group increased more at 14–15 days and 60 days (two months) of age than that of infants in the control group. Also, according to the results, breastfeeding practice was higher in mothers in the experimental group than in the control group for 14-15-day-old LBW infants.

The study findings revealed that the LBW infants’ weight increased more 14–15 days and two months after the intervention. The present study’s findings are similar to the findings of Meliat et al.‘s study in which training of mothers could influence the weight of their 0-12-month-old infants with LBW history [23]. A similar study showed that by home visitation, breastfeeding of LBW infants’ mothers was improved, and subsequently, the weight of these infants increased (17). This study is also in line with a randomized controlled trial showing that peer support education encouraged mothers in the experimental group to further implement kangaroo mother care and thus helped their LBW infants gain more weight [24]. LBW infants are more prone to health problems, and these infants may be at risk of being underweight in the early neonatal period [25]. Therefore, the nutritional needs of infants with a history of LBW must be fulfilled by mothers [16].

The study findings also showed that planned education intervention could affect the mothers’ breastfeeding of LBW infants. These findings are in line with finding of a randomized controlled clinical trial in which lactation performance of mothers of underweight preterm infants improved after a training program based on BASNEF model [26]. Our study is also similar to a quasi-experimental study with pretest-posttest design in which structured teaching programs increased mothers’ mean knowledge on breastfeeding LBW infants [27]. It can be concluded that lactation support by healthcare providers has a significant role in improving the mother’s breastfeeding practice. In this regard, a quasi-experimental study conducted in Denmark showed that training programs for neonatal nurses could improve breastfeeding performances such as skin-to-skin contact and early initiating breastfeeding of preterm mothers [19].

Mothers with LBW infants more probably experience shock, helplessness, distress, and emotional disorder than mothers with healthy newborns [28], which can affect LBW breastfeeding. In this regard, the results of a longitudinal study showed that LBW infants’ breastfeeding rates declined and were far from meeting the Healthy People 2020 ever breastfeeding goal. Thus, there was a need to plan strategies encouraging breastfeeding of LBW infants [29]. Furthermore, in a pilot project in India, 65 mother-baby dyads were assessed for seven days regarding feeding details. Evidence revealed that mothers did not have sufficient practice to initiate breastfeeding. Thus, more attention should be paid to educational issues [30].

Mothers with LBW infants have special educational needs in terms of breastfeeding. LBW infants cannot suck effectively. Thus, their mothers need to be educated for early breastfeeding initiation within six hours after childbirth and supported to follow recommendations regarding the continuation of exclusive breastfeeding up to six months [12]. Thus, making breastfeeding decisions as a significant factor can be improved by nurses through reducing barriers and supporting mothers to have successful breastfeeding [25].

One limitations of the study was that the researchers did not include preterm or very preterm LBW infants in this study. Indeed, preterm LBW infants are less likely than LBW infants to receive mother’s own milk. Furthermore, sucking ability in preterm LBW infants differs significantly from that in the term infants. Moreover, very preterm (< 32 weeks’ gestational age) or extremely preterm infants (< 28 weeks’ gestational age) poorly receive mother’s own milk by breastfeeding. Therefore, further research is recommended to be conducted on mother’s breastfeeding practice in preterm or very preterm LBW based on their sucking ability. Other limitation of the study was the intervention of other family members such as grandmothers, which might have affected the mother’s breastfeeding practice. Another limitation of the study was its relatively small sample size. Thus, future similar studies are suggested to consider taking a larger sample size. Other limitations of the study included lack of blinding, which was not possible to implement in this study. However, the statistics specialist were not aware of the allocation of participants in the study groups.

## Conclusions

The research results showed that the planned lactation education positively affected the mother’s breastfeeding practice and weight gain in LBW infants. It is thus recommended to implement planned lactation education to improve the mothers’ decisions for breastfeeding practice and consequently weight gain in their LBW infants.

## Data Availability

All data analyzed during this study are included in this published article.

## Abbreviations

LBW: Low Birth Weight
OMM: Own Mother’s Milk
MOM: Mother’s Own Milk
